# Rural and urban residents’ attitudes and preferences toward COVID-19 prevention behaviors in a midwestern community

**DOI:** 10.1371/journal.pone.0286953

**Published:** 2023-06-23

**Authors:** Laura A. Maciejko, Jean M. Fox, Michelle T. Steffens, Christi A. Patten, Hana R. Newman, Paul A. Decker, Phil Wheeler, Young J. Juhn, Chung-Il Wi, Mary Gorfine, LaPrincess Brewer, Pamela S. Sinicrope

**Affiliations:** 1 Mayo Clinic Alix School of Medicine, Rochester, MN, United States of America; 2 Department of Gastroenterology, Mayo Clinic, Rochester, MN, United States of America; 3 Department of Nursing, Mayo Clinic, Rochester, MN, United States of America; 4 Community Engagement Program, Center for Clinical and Translational Science, Mayo Clinic, Rochester, MN, United States of America; 5 Behavioral Health Research Program, Department of Psychiatry and Psychology, Mayo Clinic, Rochester, MN, United States of America; 6 Mayo Clinic Alix School of Medicine, Scottsdale, AZ, United States of America; 7 Department of Health Sciences Research, Mayo Clinic, Rochester, MN, United States of America; 8 Precision Population Science Lab, Department of Pediatric & Adolescent Medicine, Mayo Clinic, Rochester, United States of America; 9 Department of Cardiovascular Medicine, Mayo Clinic College of Medicine, Rochester, MN, United States of America; 10 Center for Health Equity and Community Engagement Research, Mayo Clinic, Rochester, MN, United States of America; University of Ilorin, NIGERIA

## Abstract

Rural populations are more vulnerable to the impacts of COVID-19 compared to their urban counterparts as they are more likely to be older, uninsured, to have more underlying medical conditions, and live further from medical care facilities. We engaged the Southeastern MN (SEMN) community (N = 7,781, 51% rural) to conduct a survey of motivators and barriers to masking to prevent COVID-19. We also assessed preferences for types of and modalities to receive education/intervention, exploring both individual and environmental factors primarily consistent with Social Cognitive Theory. Our results indicated rural compared to urban residents performed fewer COVID-19 prevention behaviors (e.g. 62% rural vs. 77% urban residents reported wearing a mask all of the time in public, p<0.001), had more negative outcome expectations for wearing a mask (e.g. 50% rural vs. 66% urban residents thought wearing a mask would help businesses stay open, p<0.001), more concerns about wearing a mask (e.g. 23% rural vs. 14% urban were very concerned about being ‘too hot’, p<0.001) and lower levels of self-efficacy for masking (e.g. 13.9±3.4 vs. 14.9±2.8, p<0.001). It appears that masking has not become a social norm in rural SEMN, with almost 50% (vs. 24% in urban residents) disagreeing with the expectation ’others in my community will wear a mask to stop the spread of Coronavirus’. Except for people (both rural and urban) who reported not being at all willing to wear a mask (7%), all others expressed interest in future education/interventions to help reduce masking barriers that utilized email and social media for delivery. Creative public health messaging consistent with SCT tailored to rural culture and norms is needed, using emails and social media with pictures and videos from role models they trust, and emphasizing education about when masks are necessary.

## Introduction

Living in a rural area has long been recognized as a source of health disparities, with higher levels of morbidity and mortality from certain chronic diseases [1–3]. Rural populations are also more vulnerable to the impacts of COVID-19 compared to their urban counterparts [4, 5]. Those living in rural communities have a higher proportion of risk factors that make them more vulnerable to the effects of COVID-19 such as: less insurance coverage, less healthcare facilities and those that include intensive care, more co-morbidities and disability, and more residents age 65 or older. Furthermore, COVID-19 incidence is higher in rural vs. urban counties since June 2021 [6, 7]. While the COVID-19 vaccine is disseminated across the U.S., as new variants emerge there remains a need to focus on maintenance and adoption of primary prevention measures, including the correct and consistent use of a 2-layer mask—a novel behavior for Americans [8]. This need is especially salient in rural communities, as shown by a poll released by the Kaiser Family Foundation (KFF) [9]. Specifically, this poll demonstrated significant hesitancy to vaccination among rural dwellers, with 35% reporting they did not intend to get a COVID-19 vaccine, even if it were deemed safe, effective, and free, as compared to 26–27% of urban and suburban dwellers [9]. As new variants emerge and spread, COVID-19 vaccination rates in rural counties lag behind those in urban counties [10, 11], with both rates being suboptimal. According to the CDC as of January 31, 2022, vaccination coverage with the first dose of the primary series was lower in rural counties compared to urban counties (58.5% vs 75.4%) [11]. Masking mandates have been used by several states in an effort to curb the spread of the virus, especially in light of evolving knowledge that vaccinated individuals can spread COVID-19 and its variants [12]. Because mask-wearing is a complex behavior that requires both individual and collective cooperation to be effective (e.g. making sure masks are worn properly), there remains a need for social and behavioral science research drawing on behavioral change principles to guide intervention development [13].

Social cognitive theory (SCT) SCT [14] provides a useful framework for understanding both individual and social-environmental level determinants of mask use. SCT emphasizes the concept of reciprocal determinism, whereby individual behavior, such as the choice to wear a mask correctly over nose and mouth in, occurs within the context and interplay of a person with their environment [14–19]. Masking behavior, collective in nature, occurs in the continually changing environment of a worldwide pandemic. To further complicate matters, government and public health leaders have not been united on the recommendation to wear a mask and further confusion is perpetuated by influential voices on social media, television, and print media [20]. The SCT construct, self-efficacy is the most proximal predictor of mask-wearing behavior, whereby those with the highest degree of confidence in their ability to perform a behavior are the ones most likely to adopt the behavior. Other key constructs within the theory include behavioral capability (having the knowledge and skill to perform the behavior), outcome expectations, a person’s understanding of what consequences will follow a behavior, social norms, or the perception that most are performing the desired behavior, and observational learning, or the ability to observe and see others perform the desired behavior [14].

Previously we conducted a community-based survey in a mixed rural-urban, southeastern Minnesota (SEMN) community (N = 7,781, 51% rural) [21]. We found that willingness to wear a mask was lower among rural residents compared to urban residents (88% vs. 95%, p<0.001). Using an SCT framework, the current study explored rural vs. urban differences in barriers to mask wearing and, among those willing to wear a mask, preferences for potential COVID-19 educational interventions. We hypothesized that rural residents would report lower levels of other COVID-19 prevention behaviors, less knowledge about COVID-19 transmission, more negative outcome expectations for wearing a mask, less distress about COVID-19, lower self-efficacy, political beliefs inconsistent with wearing a mask, and higher levels of barriers to wearing a mask compared to their urban counterparts. These data will help to tailor future education/interventions to promote masking to stop the spread of COVID-19.

## Methods

### Overview

Ethics approval was obtained and the study was deemed exempt (“minimal risk”) by the Mayo Clinic Institutional Review Board. The 26–39 question survey (length determined by branching logic) was developed using or adapting existing items from the literature, including the NIH Phen-X toolkit [22] with feedback from Mayo’s Survey Research Center (SRC) and Community Engagement in Research Advisory Board (CERAB). All study materials, including the survey questions and recruitment materials, were designed to be non-directive about masking to reduce bias related to social desirability and to be inclusive. Following the principles of community-engaged research [23], CERAB was involved at all phases of the study. Specifically, they provided feedback on the overall study design and survey (content and pilot testing) and helped promote the survey to our community. The survey, designed to meet a 6^th^-grade reading level as determined by Microsoft Word whose scale is consistent with Flesch-Kincaid reading scale [24], was programmed into Research Electronic Data Capture (RedCAP) [25] for participant ease of use. The final survey took about 15–20 minutes to complete with the following sections: 1) about wearing a mask, 2) about coronavirus, 3) about you, 4) intervention questions, and 5) final thoughts. The terms ‘mask’ and ‘Coronavirus’ were defined in the opening of the survey. Participants were not offered any incentives to complete the survey. The survey was conducted between August 4-September 4, 2020, a time when there was a state mask mandate requiring residents to wear a mask whenever in indoor public spaces and for workers who were unable to socially distance outside.

### Study design and population

To develop a survey and recruitment plan that would be inclusive and draw in widespread participation, we worked closely with CERAB and the SRC. The survey was anonymous to encourage those who might be concerned about privacy and social desirability to participate. Given the potential controversial nature of masking, we wanted respondents to feel comfortable expressing views that might contradict recommendations and/or mandates. The survey was offered to Southeastern Minnesota residents aged 18 and over from August 4-September 4, 2020. During this time, there was a state mandate to wear masks in indoor spaces and outside when social distancing was not possible. Based on rural-urban commuting area (RUCA) classification [26] using RUCA 1 and 1.1 for urban areas, SEMN is 32% urban and 68% rural.

### Outreach/Recruitment

A multifaceted approach including direct community partnerships, social media announcements, and email communications was utilized. Direct community partnerships involved Mayo Clinic internal outreach organizations and established community-based participatory research (CBPR) partnerships (Public Affairs, Employee Resource Groups, CERAB, FAITH! (Fostering African-American Improvement in Total Health) [27] as well as external community partnerships (Rochester Ready and The Center Clinic). Social media advertising was provided via posts shared on Mayo Clinic’s Facebook and Twitter pages. Extensive email outreach included contacting more than 500 businesses/groups in SEMN. All contacted organizations received an outreach email explaining the nature of the research and instructions regarding how to forward survey announcements with their respective communities. Phone and email support were provided as needed while the survey was open to public input. News and media interviews were granted upon request with approval per Mayo Clinic Public Affairs policies.

## Measures

### Socio-demographics

Gender identity, age, ethnicity/race, zip code, education level, employment status, occupation, political affiliation [28], and rural status as defined by the Rural-Urban RUCA classification [26] were assessed.

### Social cognitive theory measures

To assess COVID-19 prevention behaviors [22] (e.g. wearing a mask when out in public, staying six (6) feet away from others) we asked “In the past seven (7) days, how often did you do the following?” with 9 item measure and a 4-point Likert response from ‘none of the time’ to ‘all of the time.’ We assessed COVID-19 knowledge by asking “how does Coronavirus spread?” with participants being able to select all that apply [29]. We developed a study-specific, 10-item measure, composed of 4 attitude items (i.e. wearing a mask … makes me look weak) and 6 outcome expectations (i.e. wearing a mask… will help businesses stay open), to assess the perceived impacts of wearing a mask with a 4-point Likert response from ‘strongly disagree’ to ‘strongly agree.’ We adapted a previous measure [30] to assess masking norms (e.g. ‘I expect that most people in my community will wear a mask to stop Coronavirus’) measured by a 4-point Likert response from ‘strongly disagree’ to ‘strongly agree.’ We adapted the widely-used IES-6 [31], a brief, validated 6-item screening tool with a high degree of reliability used to assess posttraumatic stress reactions, to specifically apply to COVID-19. Respondents were asked how often they experienced distress or felt bothered about COVID-19 using a 4-point Likert response from “not at all” to “often.” To assess perceived likelihood and severity of getting COVID-19, we asked “How likely do you think it is that you could get Coronavirus?” measured by a 4-point Likert response from ‘not at all likely’ to ‘very likely” and “If you got Coronavirus, how serious do you think it would be for you?” measured by a 4-point Likert response from ‘not at all serious’ to ‘very serious.’ To assess self-efficacy, respondents were asked, “How sure are you that you could do the following skills?” (e.g., wear your mask so it covers your nose) measured by a 4-point Likert response from ‘not at all sure’ to ‘very sure.’

To assess information-seeking practices, we asked participants to select what sources (e.g., friends or family members, healthcare providers/institutions, social media platforms, news/TV/radio stations, and Government/politics) they have looked for information about Coronavirus during the previous seven days. We also assessed perceived level of trust for accurate COVID-19 information from the CDC, doctors or other healthcare providers, WHO, State/County/City Health Department, Governor/Mayor, President Trump, and official government websites using a 4-point Likert response from ‘not at all’ to ‘completely’[22]. Barriers and concerns to wearing a mask were assessed by a 12-item measure asking the level of concern about barriers to wearing a mask (e.g. foggy glasses, too hot, trouble understanding what people are saying) with a 4-point Likert response from ‘not at all concerned’ to ‘very concerned.’

## Preferences for potential education/interventions

Those who reported being somewhat willing to very willing to wear a mask were asked about their preferences for potential mask education and intervention. Respondents were asked for their topic preferences (e.g., when masks are or aren’t needed, how to care for your mask(s)…) and top choices for receiving information (e.g., by email, social media…) and were able to select all that applied. We defined a strong preference if greater than 50% of the overall sample chose an option as one of their top preference as it does not seem prudent to allocate resources to developing education or platforms for receiving interventions if it was preferred by only half or less of respondents [32].

### Statistical analysis

Data were summarized using number, percent for categorical variables; and for continuous variables we used mean, and select percentiles. Responses were compared by rural/urban status using chi-square tests (Fisher’s exact) and two-sample t-tests (rank sum) as appropriate. Missing data were excluded from analyses for the given questions. P-values <0.05 were considered statistically significant.

### Geospatial analysis

Geocoding: The zip codes of all persons surveyed during the study period were recorded as part of the survey data. Using the Census Bureau’s Zip Code Tabulation Area map [33], we were able to map other survey variables by Zip Code. Geospatial analysis was performed using ArcMap 10.4.1 [34] (produced by ESRI).

## Results

### Rural vs. urban socio-demographics

**Fig 1:**
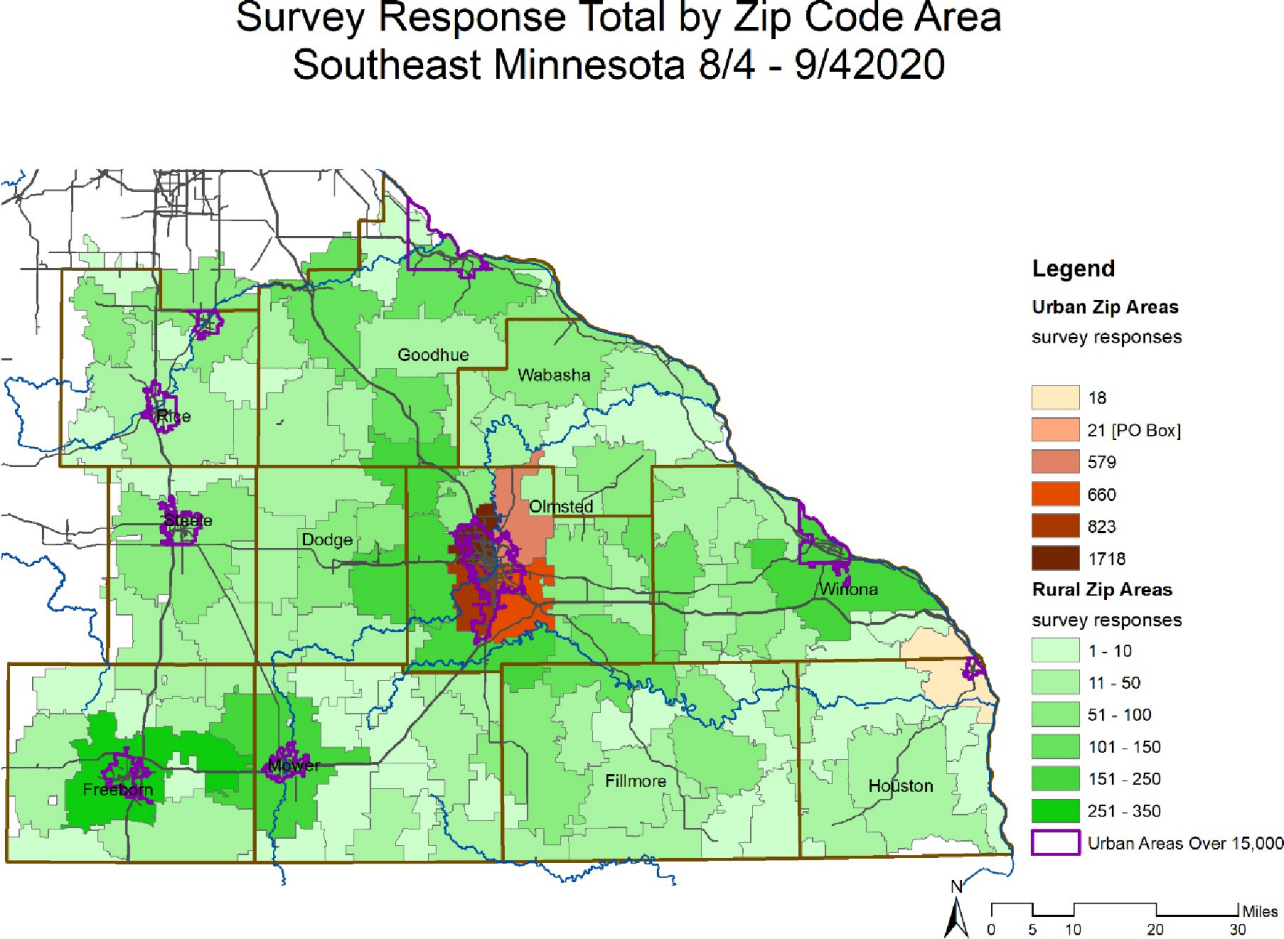
A total of 7,781 respondents across SEMN answered the online, anonymous, community-based survey. All counties eligible for the survey were represented. Of these respondents, 3963 (51%) lived in a rural area and 3,818 (49%) lived in an urban area.

Our survey had 7,781 respondents from SEMN, of which 6100 (78%) identified as female, 1521 (20%) identified as male, and 160 (2%) identified as other genders. Of these respondents, 3963 (51%) lived in a rural area while the remaining 3,818 (49%) lived in an urban area (**Fig 1**). **Table 1** provides socio-demographics overall and by rural and urban status. Fewer rural residents reported a college degree or higher education compared to urban residents (54% vs. 74%, p<0.001) and a lower percentage of rural residents reported Democrat party affiliation (33% vs. 47%, p<0.001).

**Table 1:** Demographic characteristics: Southeastern MN voluntary survey sample by rural vs. urban status, N (%).

Characteristic	Rural N = 3963	Urban N = 3818	Total	P*
Gender				0.002
Female	3167 (80)	2933 (77)	6100 (78)	
Male	713 (18)	808 (21)	1521 (20)	
Other	83 (2)	77 (2)	160 (2)	
Age				<0.001
18–29	504 (13)	603 (16)	1107 (14)	
30–39	944 (24)	1032 (27)	1976 (25)	
40–49	958 (24)	838 (22)	1796 (23)	
50–59	755 (19)	633 (17)	1388 (18)	
60–69	563 (14)	500 (13)	1063 (14)	
70–79	213 (5)	190 (5)	403 (5)	
80+	26 (1)	22 (1)	48 (1)	
Ethnicity				0.003
Non-Hispanic	3864 (98)	3692 (97)	7556 (98)	
Hispanic	74 (2)	111 (3)	185 (2)	
Race				
White	3855 (97.)	3605 (94)	7460 (96)	<0.001
Black or African American	12 (0.3)	44 (1.2)	56 (0.7)	<0.001
American Indian or Alaska Native	25 (0.6)	33 (0.9)	58 (0.7)	0.23
Asian	29 (19)	122 (3.2)	151 (1.9)	<0.001
Other	75 (1.9)	74 (1.9)	149 (1.9)	
Employment Status				<0.001
Employed	3228 (82)	3103 (81)	1832 (24)	
Employed and working outside home	2387 (60)	2098 (55)	4485 (58)	
Unemployed	729 (18)	711 (19)	1440 (19)	
College Degree	2139 (54)	2782 (74)	4921 (64)	<0.001
In politics today, what do you consider your political affiliation?				<0.001
Prefer not to share	752 (19)	500 (13)	1252 (16)	
Democrat	1283 (33)	1796 (47)	3079 (40)	
Independent	660 (17)	705 (19)	1365 (18)	
Republican	1002 (25)	581 (15)	1583 (20)	
Something else	243 (6)	223 (6)	466 (6)	

*Chi-square test (exact) or two-sample t-test (rank sum) as appropriate

### Rural vs. urban SCT-based COVID-19 behaviors and experiences

**Table 2** summarizes differences in rural vs. urban COVID-19 behaviors and experiences. When asked about COVID-19 prevention behaviors in the past 7 days, 63% of rural residents reported wearing a mask ‘all of the time’ compared to 77% of urban residents (p<0.001). Rural residents had a lower percentage of respondents answering all COVID-19 knowledge questions correctly (62% vs. 65%, p = 0.007). Rural residents had fewer respondents who ‘strongly agreed’ that masking will help businesses stay open compared to urban residents (50% vs. 66%, p<0.001). More rural residents ‘strongly agreed’ that masking should be their choice compared to urban residents (30% vs. 17%, p<0.001). Expectations that people in their community will wear a mask to stop the spread of Coronavirus varied as 11% of rural residents responded ‘strongly agree’ while 21% of urban residents responded ‘strongly agree’ (p<0.001). Rural residents reported lower levels of distress about COVID-19 compared to their urban counterparts (12.5±8.0 vs. 13.2±7.7, <0.001). Rural residents reported lower levels of self-efficacy for mask wearing behaviors (13.9±3.4 vs. 14.9±2.8, p<0.001). Both rural and urban residents reported low levels of being ‘very sure’ that they could ask someone they don’t know to put on a mask (11% and 15% respectively), and more rural residents responded ‘not at all sure’ compared to urban residents (46% vs. 34%), p<0.001.

**Table 2:** Social cognitive and behavioral factors related to COVID-19 prevention by rural vs. urban status in southeastern, Minnesota, N (%).

Characteristic	Rural N = 3963	Urban N = 3818	P*
**COVID-19 Prevention behaviors**			
Wearing a mask when out in public			<0.001
None of the time	169 (4)	85 (2)	
Some of the time	517 (13)	218 (6)	
Most of the time	801 (20)	585 (15)	
All of the time	2460 (62)	2901 (77)	
Staying six (6) feet away from others			<0.001
None of the time	223 (6)	87 (2)	
Some of the time	955 (24)	679 (18)	
Most of the time	1952 (49)	2066 (54)	
All of the time	820 (21)	972 (26)	
Hand washing or hand sanitizing			<0.001
None of the time	43 (1)	22 (1)	
Some of the time	225 (6)	136 (4)	
Most of the time	883 (22)	804 (21)	
All of the time	2784 (71)	2843 (75)	
Covering coughs and sneezes			<0.001
None of the time	38 (1)	27 (1)	
Some of the time	84 (2)	40 (1)	
Most of the time	437 (11)	364 (10)	
All of the time	3378 (86)	3345 (89)	
Not touching my face			<0.001
None of the time	262 (7)	179 (5)	
Some of the time	1225 (31)	1032 (27)	
Most of the time	1844 (47)	1857 (49)	
All of the time	600 (15)	722 (19)	
Not touching surfaces in public places			<0.001
None of the time	484 (12)	302 (8)	
Some of the time	1328 (34)	1300 (34)	
Most of the time	1543 (39)	1556 (41)	
All of the time	592 (15)	651 (17)	
Wearing gloves			<0.001
None of the time	3332 (85)	3075 (81)	
Some of the time	469 (12)	578 (15)	
Most of the time	93 (2)	79 (2)	
All of the time	47 (1)	71 (2)	
Staying at home			<0.001
None of the time	714 (18)	391 (10)	
Some of the time	1491 (38)	1307 (34)	
Most of the time	1525 (39)	1859 (49)	
All of the time	216 (5)	250 (7)	
Praying for Coronavirus to go away			<0.001
None of the time	1429 (36)	1713 (45)	
Some of the time	897 (23)	868 (23)	
Most of the time	494 (13)	391 (10)	
All of the time	1104 (28)	816 (22)	
**Knowledge about COVID-19 transmission (percent that marked true)**			
Through respiratory droplets	3788 (96)	3710 (97)	<0.001
Through close contact	3481 (88)	3497 (92)	<0.001
Through a contaminated surface	3497 (88)	3504 (92)	<0.001
Through the air	2680 (68)	2642 (69)	0.14
The virus is a hoax	153 (4)	71 (2)	<0.001
All knowledge questions correct	2456 (62)	2479 (65)	0.007
**Mask Impact Scale (Wearing a mask …)**			
Mean (SD)	21.0 (6.6)	23.7 (5.7)	<0.001
Will help businesses stay open			<0.001
Strongly Disagree	397 (10)	192 (5)	
Disagree	528 (13)	265 (7)	
Agree	1044 (27)	821 (22)	
Strongly Agree	1932 (50)	2510 (66)	
Should be my choice			<0.001
Strongly Disagree	921 (24)	1385 (37)	
Disagree	1056 (27)	1215 (33)	
Agree	749 (19)	501 (13)	
Strongly Agree	1146 (30)	637 (17)	
Should be required			<0.001
Strongly Disagree	900 (23)	468 (12)	
Disagree	560 (14)	286 (8)	
Agree	726 (19)	658 (17)	
Strongly Agree	1733 (44)	2381 (63)	
Isn’t needed			<0.001
Strongly Disagree	2100 (55)	2745 (74)	
Disagree	805 (21)	550 (15)	
Agree	582 (15)	262 (7)	
Strongly Agree	345 (9)	166 (4)	
Shows respect for others			<0.001
Strongly Disagree	439 (11)	243 (6)	
Disagree	468 (12)	209 (6)	
Agree	901 (23)	602 (16)	
Strongly Agree	2101 (54)	2731 (72)	
When others aren’t makes me feel embarrassed			0.044
Strongly Disagree	1530 (40)	1528 (41)	
Disagree	1366 (35)	1232 (33)	
Agree	747 (19)	726 (19)	
Strongly Agree	226 (6)	263 (7)	
When others aren’t makes me feel disrespected			<0.001
Strongly Disagree	1112 (29)	688 (18)	
Disagree	907 (23)	652 (17)	
Agree	1052 (27)	1212 (32)	
Strongly Agree	835 (21)	1233 (33)	
Makes me look weak			<0.001
Strongly Disagree	2508 (65)	2915 (78)	
Disagree	1124 (29)	713 (19)	
Agree	151 (4)	87 (2)	
Strongly Agree	90 (2)	40 (1)	
Makes me look threatening			<0.001
Strongly Disagree	2377 (61)	2744 (73)	
Disagree	1145 (30)	797 (21)	
Agree	238 (6)	147 (4)	
Strongly Agree	113 (3)	65 (2)	
Makes me a target for security/police			<0.001
Strongly Disagree	2470 (64)	2742 (73)	
Disagree	1146 (30)	840 (22)	
Agree	159 (4)	106 (3)	
Strongly Agree	88 (2)	60 (2)	
**Social Norm**			
I expect that most people in my community will wear a mask to stop the spread of Coronavirus			<0.001
Strongly Disagree	499 (13)	228 (6)	
Disagree	1219 (31)	683 (18)	
Agree	1785 (45)	2095 (55)	
Strongly Agree	425 (11)	787 (21)	
**Impact of Events (stress about coronavirus)**			
Mean (SD)	12.5 (8.0)	13.2 (7.7)	<0.001
I thought about Coronavirus when I didn’t mean to			<0.001
Not at all	811 (21)	618 (16)	
Rarely	770 (20)	766 (20)	
Sometimes	1400 (36)	1419 (37)	
Often	964 (24)	994 (26)	
I felt watchful or on guard			<0.001
Not at all	909 (23)	693 (18)	
Rarely	786 (20)	676 (18)	
Sometimes	1324 (34)	1423 (38)	
Often	914 (23)	1003 (26)	
Other things kept making me think about Coronavirus			<0.001
Not at all	784 (20)	605 (16)	
Rarely	734 (19)	736 (20)	
Sometimes	1431 (36)	1425 (38)	
Often	977 (25)	1017 (27)	
I was aware that I still had feelings about Coronavirus, but I didn’t deal with them			<0.001
Not at all	1523 (39)	1292 (34)	
Rarely	1183 (30)	1266 (33)	
Sometimes	904 (23)	917 (24)	
Often	309 (8)	317 (8)	
I tried not to think about Coronavirus			<0.001
Not at all	899 (23)	824 (22)	
Rarely	792 [31]	878 (23)	
Sometimes	1436 (37)	1434 (38)	
Often	800 (20)	660 (17)	
I had trouble concentrating			0.006
Not at all	1597 (41)	1411 (37)	
Rarely	1050 (27)	1075 (28)	
Sometimes	871 (22)	927 (24)	
Often	410 (10)	379 (10)	
**Perceived Likelihood of getting Coronavirus**			<0.001
I have had Coronavirus	38 (1)	18 (1)	
Not at all likely	461 (12)	372 (10)	
Somewhat likely	2118 (54)	2105 (55)	
Likely	892 (23)	955 (25)	
Very likely	447 (11)	362 (10)	
**Perceived Severity of getting Coronavirus**			<0.001
Not at all serious	1221 (31)	890 (24)	
Somewhat serious	1687 (43)	1796 (48)	
Serious	620 (16)	704 (19)	
Very serious	391 (10)	392 (10)	
**Self**-efficacy (How sure are you that you could…)^**^			
Mean (SD)	13.9 (3.4)	14.9 (2.8)	<0.001
Wear your mask so it covers your nose			<0.001
Not at all sure	42 (1)	21 (1)	
Somewhat sure	100 (3)	47 (1)	
Sure	411 (12)	282 (8)	
Very sure	2900 (84)	3243 (90)	
Wear your mask so it covers your mouth			<0.001
Not at all sure	16 (1)	10 (0)	
Somewhat sure	59 (2)	25 (1)	
Sure	428 (12)	279 (8)	
Very sure	2950 (85)	3273 (91)	
Wear your mask even when people around you aren’t wearing theirs			<0.001
Not at all sure	228 (7)	118 (3)	
Somewhat sure	315 (9)	205 (6)	
Sure	687 (20)	586 (16)	
Very sure	2220 (64)	2664 (75)	
Wear your mask for less than an hour			<0.001
Not at all sure	160 (5)	139 (4)	
Somewhat sure	138 (4)	77 (2)	
Sure	560 (17)	375 (11)	
Very sure	2545 (75)	2961 (83)	
Wear your mask for more than an hour			<0.001
Not at all sure	276 (8)	143 (4)	
Somewhat sure	311 (9)	209 (6)	
Sure	639 (19)	510 (14)	
Very sure	2204 (64)	2714 (76)	
How sure are you that you could ask someone you don’t know to put on their mask when they are around you			<0.001
Not at all sure	1578 (46)	1224 (34)	
Somewhat sure	983 (29)	1207 (34)	
Sure	498 (14)	610 (17)	
Very sure	395 (11)	550 (15)	
**Where people got information about COVID-19 in the past seven days**			
Center for Disease Control (CDC)	2065 (52)	2242 (59)	<0.001
Internet	1800 (45)	1895 (50)	<0.001
Healthcare providers	1585 (40)	1806 (47)	<0.001
News/TV/radio	1581 (40)	1729 (45)	<0.001
Healthcare organizations	1398 (35)	1813 (48)	<0.001
World Health Organization (WHO)	958 (24)	1175 (31)	<0.001
Coworkers or classmates	713 (18)	667 (18)	0.55
Government/politics	553 (14)	579 (15)	0.13
My religious/spiritual leader	89 (2)	74 (2)	0.34
**Perceived Trust for COVID-19 information**			
Centers for Disease Control (CDC)			<0.001
Not at all	402 (10)	192 (5)	
Somewhat	772 (20)	519 (14)	
Mostly	1466 (37)	1600 (42)	
Completely	1306 (33)	1494 (39)	
Doctors or other healthcare providers			<0.001
Not at all	110 (3)	52 (1)	
Somewhat	616 (16)	286 (8)	
Mostly	1699 (43)	1604 (42)	
Completely	1511 (38)	1864 (49)	
World Health Organizations (WHO)			<0.001
Not at all	745 (19)	374 (10)	
Somewhat	798 (20)	624 (17)	
Mostly	1320 (34)	1489 (39)	
Completely	1056 (27)	1300 (34)	
State, County, or City Health Department			<0.001
Not at all	323 (8)	186 (5)	
Somewhat	865 (22)	523 (14)	
Mostly	1595 (41)	1626 (43)	
Completely	1158 (29)	1461 (38)	
Governor/Mayor			<0.001
Not at all	1047 (27)	545 (14)	
Somewhat	942 (24)	798 (21)	
Mostly	1218 (31)	1539 (41)	
Completely	725 (18)	905 (24)	
President Trump			<0.001
Not at all	2387 (61)	2901 (76)	
Somewhat	814 (21)	518 (14)	
Mostly	537 (14)	271 (7)	
Completely	197 (5)	102 (3)	
Official Government Websites			<0.001
Not at all	853 (22)	613 (16)	
Somewhat	1700 (43)	1771 (47)	
Mostly	1120 (29)	1163 (31)	
Completely	238 (6)	240 (6)	

*Chi-square test (exact) or two-sample t-test (rank sum) as appropriate

** these questions were only answered by those who responded they were ‘somewhat’ to ‘very’ willing to wear a mask.

When asked where respondents received information about COVID-19 in the past 7 days, rural respondents were most likely to receive their information from the CDC, the internet, or from healthcare providers. A smaller percentage of rural residents compared to urban residents reported seeking information from healthcare organizations (35% vs. 48%, p<0.001), healthcare providers (40% vs. 47%, p<0.001), CDC (52% vs. 59%, p<0.001), WHO (24% vs. 31%, p<0.001), the internet (45% vs. 50%, p<0.001), and news/TV/radio (40% vs. 45%, p<0.001). Rural residents reported lower levels of trust in all information sources except for President Trump (39% vs 24%, p<0.001).

### Rural vs. urban concerns about wearing a mask

Among both rural and urban respondents, the top three concerns about wearing a mask included feeling ‘too hot,’ ‘foggy glasses,’ and ‘trouble understanding what people are saying.’ 23% of rural residents reported being very concerned about being ‘too hot,’ 23% were very concerned about ‘foggy glasses,’ and 26% were very concerned about ‘trouble understanding what people are saying’ compared to 14%, 17% and 15% in urban respondents (p<0.001).

### Rural vs. urban preferences for potential education/interventions

**Table 3** summarizes preferences for potential education/intervention topics, information delivery, and learning by rural vs. urban status among those willing to wear a mask. Rural residents reported interest in fewer topics compared to their urban counterparts. Top choices included ‘how to care for your mask(s)’ (62% rural vs 69% urban, p<0.001), ‘how to wear a mask (e.g., for fit, comfort…)’ (58% vs. 65%, p<0.001), and ‘how masks work’ (54% vs. 58%, p = 0.001). Top choices for receiving information were similar between the two groups, with the top two learning preferences for both rural and urban residents being email and social media. Most rural residents selected email as their top choice (59%), but social media was a close second (58%). Most urban residents selected social media as their top choice (63%), closely followed by email (62%).

**Table 3:** Preferences for potential COVID-19 education/intervention topics and delivery by rural vs. urban status in southeastern, Minnesota, N (%).

Characteristic	Rural N = 3483	Urban N = 3615	P*
**Topic preferences** **			
When masks are or aren’t needed	2317 (67)	2480 (69)	0.061
How to care for your mask(s)	2173 (62)	2478 (69)	<0.001
How to wear a mask (e.g. for fit, comfort. . .)	2028 (58)	2331 (65)	<0.001
How masks work	1886 (54)	2093 (58)	0.001
How wearing a mask will affect your medical condition	1742 (50)	1826 (51)	0.68
How to talk with others about wearing a mask	1691 (49)	2151 (60)	<0.001
Where to use a mask	1540 (44)	1730 (48)	0.002
**Top choice for receiving info** **			
Email	2046 (59)	2222 (62)	0.019
Social media (e.g. Facebook, Instagram, TikTok, Twitter. . .)	2022 (58)	2277 (63)	<0.001
Mail	990 (28)	975 (27)	0.17
In person	689 (20)	699 (19)	0.64
At places I visit (e.g. church, grocery store, gym…)	679 (20)	811 (22)	0.002
Video conference (e.g. Zoom, FaceTime, WhatsApp. . .)	446 (13)	520 (14)	0.052
Phone	193 (6)	190 (5)	0.60

*Chi-square test (exact) or two-sample t-test (rank sum) as appropriate

**these questions were only answered by those who responded they were ‘somewhat’ to ‘very’ willing to wear a mask.

## Discussion

We previously reported that urban residents were more likely to be ‘very willing’ to wear a mask to stop the spread of COVID-19 compared to rural residents (OR = 1.23, 95% CI 1.05–1.44 [21]. The current report adds knowledge on rural-urban differences on SCT-based constructs related to mask wearing. Rural residents reported more negative outcome expectations for wearing a mask (i.e. wearing a mask will not help businesses stay open) and more concerns about wearing a mask compared to their urban counterparts. Results suggest opportunities for tailoring future educational interventions. Creative messaging on a public health level is needed to reach rural residents, using emails and social media with pictures and videos relevant to rural culture and norms, and emphasizing a variety of topics about masks including when masks are necessary or not needed.

Our results indicate rural residents had lower levels of self-efficacy for masking. Self-efficacy, according to Bandura, is the single most important proximal predictor of behavior [14]. There were also differences in outcome expectations for wearing masks, i.e., fewer rural residents thought that wearing a mask would help businesses stay open (50% vs. 66%). Therefore, there are opportunities for future educational interventions among rural populations focused on how mask-wearing prevents the spread of the virus and can keep businesses open, and also on how to wear a mask to improve its effectiveness (i.e., keep the nose covered). It appears that masking has not become a social norm in the United States, especially among rural residents with almost 50% disagreeing with the expectation ’others in my community will wear a mask to stop the spread of Coronavirus’ (vs. 24% in urban residents). Despite more isolation, social influence is an important factor in rural communities, whereby members are more likely to know each other and to exert behavioral expectations or pressure to conform [35]. Therefore, if a behavior, such as masking is not perceived as a behavioral norm in a community, it may be more difficult to adopt the behavior or adoption of the behavior could result in stress and worry about being the subject of undue attention. Potential education/interventions [36] include providing free masks, providing information, and effective modeling by community or political figures, to normalize masking in both rural and urban communities.

It appears that it may be difficult for respondents to ask others to put on their masks, as seen by both rural and urban residents being ‘not at all sure’ about asking someone they don’t know to put on their mask (46% and 34%, respectively). Rural residents expressed less interest in learning how to talk to others about wearing a mask (49% vs. 60%). While both groups have that concern, rural residents express lower levels of interest in education to mitigate that concern, possibly due to interactions occurring in a less populous area (i.e., more room for social distancing). This suggests that the suboptimal mask use in rural residents might not only be due to outcome expectations but also due to behavioral capability for wearing a mask. Interventions on both the benefits of wearing a mask for the community and how to wear a mask while minimizing physical concerns will be important, especially for rural residents.

While the CDC was most widely listed for providing COVID-19 information by rural residents, only a small percentage of rural residents said that they trust the CDC completely for information about COVID-19. This finding suggests that being a reputable governmental source for healthcare information does not equate to being a trusted source. Healthcare providers were also high on the list, and though rural residents reported more trust in healthcare providers, there was also a low percentage that responded they trust healthcare workers ’completely’ for COVID-19 information. Given the mismatch between information seeking and level of trust, interventions to increase trust in these sources of information could be beneficial for rural residents. This finding is also relevant to COVID vaccination uptake. A study by Viskupič et al found that those who do not trust the CDC or healthcare providers are less likely to get vaccinated [37]. Given many of the current health interventions/marketing comes from the healthcare sector, a different approach may be more suited for rural residents. It is important to note that rural residents reported more trust in Trump compared to urban residents. This is also consistent with the fact that more rural residents reported being Republican. For this reason, we need to recognize that rural residents may be less likely to trust information coming from the Democratic administration. In other words, interventions to normalize masking that utilize relatable role models that rural residents trust may be a more effective strategy.

Improving knowledge about COVID-19 transmission might be helpful for rural residents, and increase their likelihood of wearing a mask or getting vaccinated. Top choices (>50%) for receiving information included email and social media for both rural and urban residents, which highlights the importance of focusing our efforts on online interventions.

Our work contributes to the body of research about COVID-19 prevention and masking to prevent respiratory illness and provides direction for developing interventions, especially for rural populations. This work will be especially important as we continue to promote vaccinations, which is happening more slowly in rural areas, and with less uptake than in urban areas [10], as spread of the virus must still be prevented using simple and effective measures such as hand washing, masking, and social distancing.

### Limitations

Our study has some limitations. First, our response rates for racial and ethnic minority groups (including Black/African Americans and Hispanics) were quite low despite extensive community outreach. While we met repeatedly with our CERAB from the inception of the study idea onward, involvement of even more community leaders and key community-based organizations serving these communities could have enhanced our community outreach efforts and diversified our sample. This might also be due, in part, to the constraints on our ability to engage with underserved communities through in-person outreach due to social distancing measures to prevent COVID-19 transmission. Further efforts are needed to encourage participation from these members of our community to ensure that their specific needs are met by public health strategies to prevent the spread of COVID-19.

Second, while we tried to encourage all respondents to share their beliefs about masking, those who were less willing to wear a mask might have been less interested in participating, thus potentially leading to inclusion bias. Because of this, the results should be interpreted with caution as the actual willingness to wear a mask could be lower than what we reported. We attempted to minimize inclusion bias by 1) developing the survey with continual feedback from CERAB, with the goal of including non-directive language, 2) utilizing a wide range of community contacts from across the region to ensure representation across our survey area (Fig 1), and 3) making the survey anonymous to ensure respondents would feel comfortable being honest about mask wearing beliefs regardless of willingness to wear a mask. We recognize that having an online survey limits participation to those who have access to technology. And while we reported a larger response from those identifying as female, previous research [38] suggests that females are more responsive to surveys. Despite the potential for bias, our large sample size included adequate numbers in all groups analyzed.

Lastly, while we used existing measures from the Phen-X database, we also created new measures with little time to develop and test them, due to the rapidly changing circumstances of the pandemic. Despite this, we modeled our measures after existing ones, used sound survey design principles and incorporated our theoretical framework to help guide the questions with feedback from the Survey Research Center and our Community-engaged partners.

In conclusion, creating public health messaging that utilizes images and videos from role models and organizations that rural people trust and identify with and delivery those messages via email and social media will be critical to improving mask utilization as we continue to face the COVID-19 pandemic.

## Supporting information

S1 Raw data (XLSX)
